# On the Determination of Forming Limits in Polycarbonate Sheets

**DOI:** 10.3390/ma13040928

**Published:** 2020-02-19

**Authors:** Ana Rosa-Sainz, Gabriel Centeno, Maria Beatriz Silva, Jose Andrés López-Fernández, Andrés Jesus Martínez-Donaire, Carpoforo Vallellano

**Affiliations:** 1 Department of Mechanical and Manufacturing Engineering, University of Seville, Camino de los Descubrimientos s/n, 41092 Sevilla, Spain; arosa@us.es (A.R.-S.); gaceba@us.es (G.C.); jlopez85@us.es (J.A.L.-F.); ajmd@us.es (A.J.M.-D.); carpofor@us.es (C.V.); 2 IDMEC, Instituto Superior Técnico, Universidade de Lisboa, Av. Rovisco Pais, 1049-001 Lisboa, Portugal

**Keywords:** polymer sheet forming, formability limits, necking, fracture, polycarbonate

## Abstract

By proposing an adaptation of the methodology usually used in metal forming, this paper aims to provide a general procedure for determining the forming limits, by necking and fracture, of polymeric sheet. The experimental work was performed by means of Nakajima specimens with different geometries to allow to obtain strains in the tensile, plane, biaxial and equibiaxial states for Polycarbonate sheet with 1 mm of thickness. The application of the time-dependent and flat-valley approaches used in metals has been revealed appropriate to characterize the onset of necking and obtain the forming limits of polycarbonate, despite the stable necking propagation typical of polymeric sheets. An analysis of the evolution of the strain paths along a section perpendicular to the crack allowed for a deeper understanding of the steady necking propagation behaviour and the adoption of the methodology of metals to polymers. The determination of the fracture strains was enhanced with the consideration of the principal strains of the DIC system in the last stage, just before fracture, due to the significant elastic recovery typical of polymeric sheets. As a result of this analysis, accurate formability limits by necking and fracture are obtained for polycarbonate sheet, together with the principal strain space, providing a general framework for analysing incremental sheet forming processes where the knowledge of the fracture limits is relevant.

## 1. Introduction

Polymers represent a significant part of the raw materials used nowadays in the manufacturing industry. This happens, due to their lightweight, high corrosion resistance, good thermal and electrical isolation properties, among other advantages.

The shaping of polymers into a final part is commonly performed by means of thermal operations involving heating, forming and cooling. It is only economically viable for mass production, due to the high costs with tools design and manufacturing. Examples of these melt-based manufacturing processes are extrusion, moulding, casting and thermoforming [1].

Recently, the current industry trend for low volume, lightweight, high quality and customization of products has led to the development of more flexible manufacturing processes, with short forming cycles, short development and production times. These new manufacturing processes are compatible with rapid prototyping and small-batch production.

Traditional prototyping involved machining, joining and simple moulding [1]. Later other processes were developed to allow the faster manufacturing of prototypes. These processes also known as additive manufacturing, where the parts are manufactured layer by layer, allows the industry to obtain customized parts with complex geometry, in a relatively short time and low cost [2,3].

An alternative to the additive manufacturing processing of polymers was proposed by Franzen et al. [4] which presented the manufacturing of polymeric sheet parts by incremental forming performed at room temperature. This process consists in the deformation of a sheet clamped in a rig that is deformed by a series of incremental deformations imposed by a universal tool, attached to a CNC machine or robotic arm, which follows the desired contour of the part. 

Cold forming of polymers points to the mid-1960s, where several conventional sheet and bulk metal forming operations were applied to cold plastic deformation of polymers. The review publication of Shaw [5] identifies the most relevant ones. Some advantages of cold forming of polymers were identified with these works, such as short time cycles, less energy, better mechanical properties and simpler and less expensive tooling. In parallel, the need to characterize the polymer’s behaviour in cold forming emerged. Such as the pioneering pressure-dependent yield criteria for polymers by Caddell et al. [6].

Lately, the application of single point incremental forming process to polymeric sheet has raised the interest for cold forming of polymers, its formability and failure. This interest emerged with the experimental verification that strains of incremental formed sheet metals were far above the formability limit by necking or forming limit curve (FLC) [7]. This behaviour was later also confirmed for polymers by Silva et al. [8], Alkas Yonan et al. [9] and Bagudanch et al. [10].

In this sense, Marques et al. [11] proposed an experimental methodology to determine the formability limits for four different thermoplastics sheets: Polyamide (PA), polycarbonate (PC), polyethylene terephthalate (PET) and polyvinylchloride (PVC). The methodology made use of circle grid analysis (CGA) for the determination of necking limit (or FLC) and for the fracture thickness measurements to obtain the ‘gauge length’ strains of fracture limit line (FFL), similarly to the methodology used for sheet metal [12]. Results revealed that the FLC and the FFL should be considered as a unique limit. However, the CGA is not the best methodology to determine the necking limit, due to the typical stable neck propagation of polymers after the onset of necking, as pointed out by Carothers and Hill [13].

Several researchers studied the influence of process parameters in single point incremental forming (SPIF) of polymers, for example, geometry [14], force and temperature [15,16], rotation of the tool and step down [17], friction between the tool and workpiece [18] and tool diameter [19] among others. The optimization of several parameters, to reduce the springback of the final part [20], and to minimize energy consumption and costs [21], were also analysed.

The medical application of SPIF of polymeric sheets to cranial prostheses has been investigated by Bagudanch et al. [22], Centeno et al. [23] and Clavijo-Chaparro et al. [24]. New approaches of SPIF in hot conditions were also recently developed by Ambrogio et al. [25] and Al-Obaidi et al. [26].

These new developments and trends are giving rise to the need for knowledge of polymeric sheet behaviour. In this regard, Alkas Yonan et al. [27] developed a material model that considers the non-linear material behaviour in loading and unloading, which was validated for high-density polyethylene plastic (HDPE), polyvinyl chloride (PVC) and polycarbonate (PC). Bagudanch et al. [28] analysed the constitutive equations for polyvinyl chloride (PVC) and polycarbonate (PC) by means of tensile tests at several temperatures and testing speeds. Recently, Ye et al. [29] analysed the necking propagation, for different strain rates of tensile test specimens, by means of a digital image correlation (DIC) system.

Thus, this work revisits the methodology for the determination of formability limits by necking and by fracture of polymers, proposing an adaptation of the sheet metal forming procedures [12]. The experimental work is performed by means of Nakajima tests with four different geometries to scan the strain paths from pure tension until equibiaxial expansion of PC sheets with 1 mm of thickness at room temperature. The aim of this work is to investigate the feasibility of this methodology to obtain the formability limits accurately.

## 2. Experimentation

The investigation was carried out on Polycarbonate (PC) sheets with 1 mm of thickness, obtained by extrusion. The determination of the formability limits by necking and by fracture of the PC sheets was performed by means of Nakajima tests with a tool following the standard ISO 12004-2 [30] on a universal sheet testing machine (model 142-20, Erichsen, Germany) at room temperature (25 °C).

The specimens were machined out from the supplied sheets. Four different geometries were considered in order to achieve different strain loading paths, from tensile strain towards equibiaxial strain. The specimens’ geometry was aligned longitudinally with the extrusion direction of the sheet.

Table 1 presents the experimental work plan with geometry and operation parameters for each condition: Tensile strain (TS), plane strain (PS), biaxial strain (BS) and equibiaxial strain (EBS) specimens. In order to ensure reproducibility of the results, tests were performed in random order, and three repetitions were made for each specimen geometry. In order to minimize the friction, between the punch and the polymeric specimens, one layer of Polytetrafluoroethylene (PTFE) was sandwiched with two layers of Vaseline.

The strain measurements on the deformation area, on the vicinity of the zones where cracks are opened, were performed using the digital image correlation (DIC) system (ARAMIS-v6.2.0-6, GOM, Germany). The Aramis system was equipped with 2 CCD cameras with an angle between them of 23.6° and lens with 50 mm of focal length. The frequency of image acquirement was set to 12 frames per second and with a facet size of 13 × 11 pixel. A black and white stochastic pattern on the specimens’ surface was defined by a fine spray of matte black on a white background.

## 3. Methodology

This investigation is aimed at providing a methodology for the determination of the formability limits by necking and fracture in principal strain space for polymeric sheets. This methodology is a first attempt to adapt to polymeric sheet the methodologies commonly applied for metals [12,31].

### 3.1. Forming Limit by Necking

Necking is a failure mode that occurs by tension where relatively large amounts of strain are located disproportionately in a small region of the material. Two different approaches are considered and adapted for the determination of the onset of necking for PC sheets: A time-dependent and a time-position dependent (also known as “flat-valley”) originally proposed by the authors [31].

#### 3.1.1. Time-Dependent Methodology

**Figure 1:**
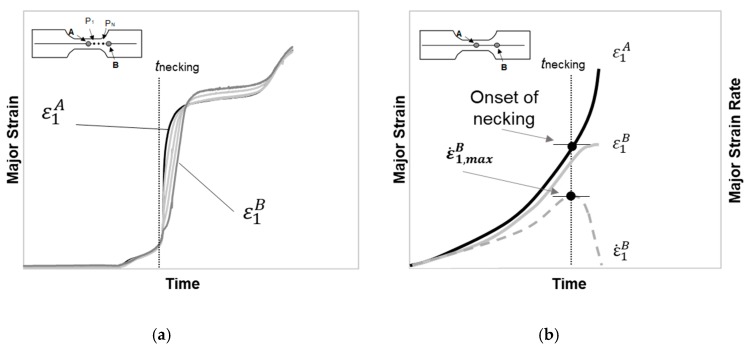
Schematic representation of the major strain and major strain rate evolution with time for a polycarbonate Nakajima test with pure tension geometry. (**a**) Evolution of major strains with time for the necking region, (**b**) Time-dependent approach application, adapted from Martínez-Donaire et al. [31].

Figure 1 presents a schematic evolution of the major strain and major strain rate with time for a pure tension geometry Nakajima test in PC. The deformation in polymers is homogeneous along with the specimen until a certain area reduction, and local reduction in thickness is achieved. Later, the neck propagates steadily along the length of the specimen and until fracture [32].

The time-dependent approach for metals is based on the experimental evidence of the necking initiation and its development [31]. This approach makes use of the major strain temporal analysis and its first-time derivative or major strain rate, ${\dot{\varepsilon }}_{1}$, for a series of points along a section perpendicular to the crack. The contrast between the localized thinning characteristic of the metals and the necking behaviour of polymers required some adaptation of the original approach.

The identification of the onset of necking is of major importance to the application of the time-dependent approach because polymers neck propagation starts on a different cross-section than the fracture cross-section of the specimen. Figure 1a shows the representation of the major strain with time for several points along a section perpendicular to the site of necking initiation (points A, P_1_, …, P_N_, B), this representation allows to identify the two reference points at the necking zone: Point A and point B. Point A corresponds to the point where the necking initiates, the points between points A and B, also have a monotonically increase of strains and point B is the first point where the strain starts ceasing (see Figure 1a,b) temporarily identifying the boundary of the instability region.

Figure 1b shows a schematic representation of the major strain evolution of point A and B with time and the strain rate evolution of point B with time. The onset of necking is established when the strain rate for point B reaches a local maximum, ${\dot{\varepsilon }}_{1,max}^{B}$, that time instant is denoted $t_{necking}$. Thus, the limit strains at the onset of necking will correspond to the strains for point A $\left(\varepsilon_{1,lim},\varepsilon_{2,lim}\right)$ at the time instant $t_{necking}$.

#### 3.1.2. Flat-Valley Approach

The flat-valley approach is based on the direct observation and analysis of the displacements of the outer surface of the sheet during necking [31].

**Figure 2:**
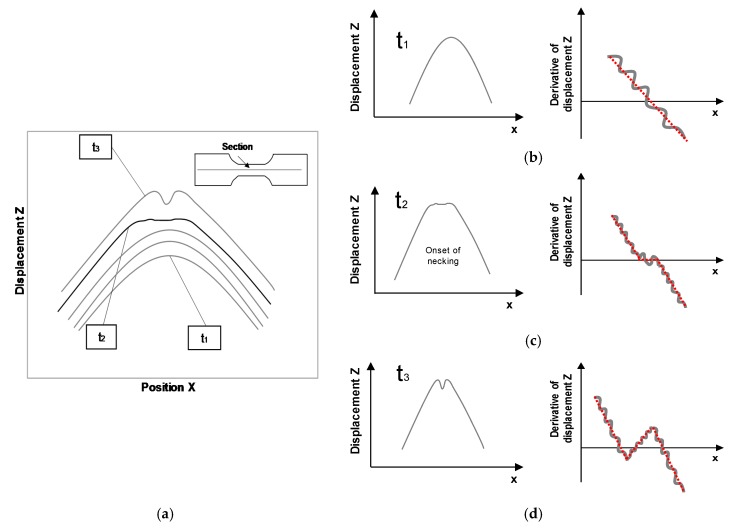
Schematic representation along a section perpendicular to the failure area of a Nakajima test. (**a**) Z-displacement over the X-position. Temporal evolution of the Z-displacement and its spatial derivative on a section perpendicular to the failure area at an instant: (**b**) Before necking, (**c**) onset of necking and (**d**) after necking. Adapted from Martínez-Donaire et al. [31].

Figure 2a shows the schematic evolution of the Z-displacement (normal direction to the initial surface) over the X-position along a section perpendicular to the necking area of a Nakajima test, and each profile corresponds to an instant of time. The time is identified by the stages obtained by the DIC system, that correspond directly to the frequency of acquisition. The profile evolution before the onset of necking follows the punch geometry (stage **t_1_** in Figure 2). Then the onset of necking stage reveals a flat profile or flat-valley (stage **t_2_** in Figure 2) corresponding to the inability of the sheet to follow the punch geometry as in the previous stage (stage **t_1_** in Figure 2). After the time instant of the onset of necking the valley becomes clearly visible (stage **t_3_** in Figure 2a). In polymers, this identification is sometimes difficult, since the onset of necking and fracture do not occur for the same cross-section, due to the necking propagation process along the specimen length.

Figure 2b–d represent the temporal evolution of the Z-displacement profile and its spatial derivative for three different stages, or time instants, of the same section perpendicular to the necking area. An alternative way to identify the instant of the onset of necking is to consider the first spatial derivative of the Z-displacement, for which when its slope remains locally constant corresponds to the typical flat profile at the onset of necking.

### 3.2. Forming Limit by Fracture

The procedure to determine the formability limits by fracture is based on the measurement of the fracture thickness from the fractographies of the fractured specimens [12].

The logarithmic strain in the thickness direction is calculated as follows,

(1) $$\varepsilon_{3f}=\ln\frac{t_{f}}{t_{0}}$$

where $t_{f}$ is the current sheet thickness at fracture and $t_{0}$ is the initial sheet thickness.

The general application of this methodology to metals considers that the fracture minor strain variation/gradient in the sheet surface is practically null after necking [33], and it can be estimated as approximately the same as the necking one, which is,

(2) $$\varepsilon_{2f}≅\varepsilon_{2n}$$

Finally, the fracture major strain is obtained assuming volume constancy [33],

(3) $$\varepsilon_{1f}+\;\varepsilon_{2f}+\;\varepsilon_{3f}=0\underset{}{\Leftrightarrow }\varepsilon_{1f}=-\varepsilon_{2f}-\varepsilon_{3f}$$

In polymers, due to neck propagation along the length of the specimen, the original methodology developed for determining the fracture forming limit for sheet metal [12] needs to be adjusted for the polymers’ behaviour. In this sense, it is more accurate to consider that the minor strain at fracture $\varepsilon_{2f}^{*}$ is obtained considering that the local strain loading path slope $\beta^{*}$ remains constant. The slope$\;\beta^{*}$ is obtained at the last measured strains by the DIC system $\left(\varepsilon_{1}^{DIC},\;\varepsilon_{2}^{DIC}\right)$,

(4) $$\beta^{*}=\frac{d\varepsilon_{2}^{DIC}}{d\varepsilon_{1}^{DIC}}$$

The local major strain of fracture $\varepsilon_{1f}^{*}$ is calculated from the thickness reduction at fracture (1) and assuming a constant slope previously calculated (4),

(5) $$\varepsilon_{1f}^{*}=\frac{-\varepsilon_{3f}^{}}{1+\beta^{*}}$$

And the minor strain at fracture can be obtained by,

(6) $$\varepsilon_{2f}^{*}=\beta^{*}\cdot \varepsilon_{1f}^{*}$$

Finally, the limit strain pair at fracture will correspond to $\left(\varepsilon_{1f}^{*},\varepsilon_{2f}^{*}\right)$, and it will allow representing the formability limit by fracture.

## 4. Results and Discussion

This section presents the determination of the principal strain pairs at the onset of necking and fracture, of the experimental plan considered, for commercial Polycarbonate sheets with 1 mm of thickness by means of the methodology presented in Section 3. These principal strain states allow defining the formability limits by necking (FLC) and by fracture (FFL). The evolution of the strain path along a section perpendicular to the crack of the Nakajima specimens was analysed.

### 4.1. Formability Limit by Necking

The methodology described in Section 3.1 considered two different approaches to determine the strain pairs at the onset of necking, the time-dependent and the flat-valley approach [31]. These two approaches were applied to PC sheet with 1 mm of thickness in four different Nakajima specimen geometries to obtain strain paths of tensile strain (TS), plane strain (PS), biaxial strain (BS) and equi-biaxial strain (EBS). As far as the authors are aware, this was the first time that strains pairs at the onset of necking for polymers were determined by these approaches typical of sheet metal forming.

**Figure 3:**
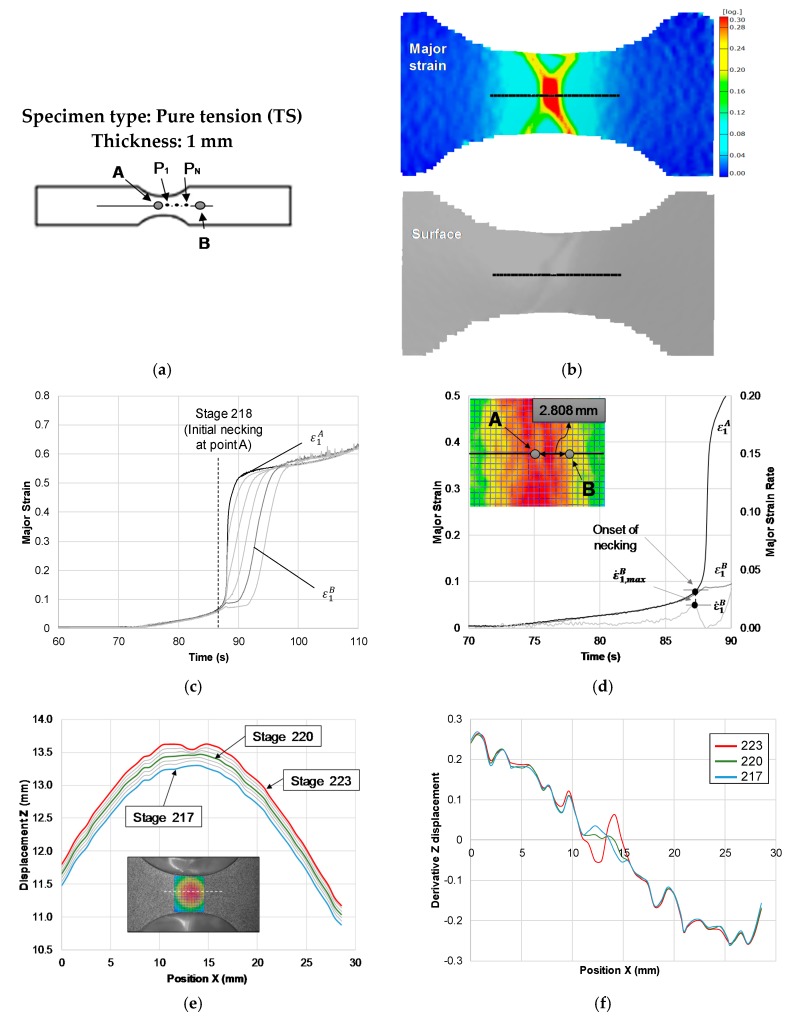
Application of the methodology to obtain the onset of necking strains for a tensile strain (TS) Nakajima specimen of PC with 1 mm of thickness. (**a**) The geometry of the specimens and identification of the section perpendicular to the failure region and selected points along its length. (**b**) Major strain (left) and surface shape (right) of the specimens’ failure area obtained by the DIC system. (**c**) Experimental time evolution of the major strain along the points of the selected section. (**d**) Time-dependent approach application. (**e**) Z-displacement along the selected section. (**f**) The spatial derivative of Z-displacement along the selected section.

Figure 3 presents the application of the two approaches to the TS Nakajima specimen of PC with 1 mm of thickness. The time-dependent approach allowed to identify point A and B that define the instability region (Figure 3a), where point B corresponds to the first point where the strain starts ceasing (Figure 3c). The evolution of the strain rate for point B is presented in Figure 3d, and it allows for a clear identification of its local maximum ${\dot{\varepsilon }}_{1,max}^{B}$, and thus, the time instant at the onset of necking $t_{necking}$. This approach allowed to obtain the limit strains at the onset of necking that correspond to point A strains $\left(\varepsilon_{1,lim},\varepsilon_{2,lim}\right)$ at the time instant $t_{necking}$.

The flat-valley approach applied to the TS Nakajima specimen of PC with 1 mm of thickness is presented in Figure 3. The Z-displacement along a section perpendicular to the necking region is presented in Figure 3e and allowed to identify the instant at which the onset of necking initiates (highlighted in green), where the thickness of the central region reduces faster than the adjacent regions and a flatten profile is visible, corresponding to the onset of necking. The spatial derivative of the Z-displacement along the selected section revealed the correspondence for the same instant the occurrence of a greater flatness confirming the onset of necking stage (Figure 3f highlighted in green), and a sign change for the stages before (Figure 3f highlighted in blue) and after (Figure 3f highlighted in red) the onset of necking stage. Table 2 presents the onset of necking strains obtained for all the specimens of the experimental plan. The results presented in Figure 3 are related with specimen II of the TS geometry, and as we can see from the results that the time-dependent methodology identified the onset of necking stage 218 and the flat-valley stage 220. This difference is negligible, due to the fact that the time interval between each stage is 1/12 s, directly related to the frequency of acquisition of 12 frames per second of the DIC system. The results for the other two TS specimens (Table 2) revealed the repeatability of the onset of necking determination for this specimen’s geometry with a maximum time difference between the two methodologies of 1/6 s. 

The application of the two approaches to the PS Nakajima specimens of PC with 1 mm thickness (Table 2) revealed appropriate to obtain the limit strains at the onset of necking as for the TS Nakajima specimen presented in Figure 3. The maximum difference verified in terms of stages number was 3, which revealed the repeatability of the onset of necking determination for this specimen’s geometry with a maximum time difference between the two methodologies of 1/4 s. 

**Figure 4:**
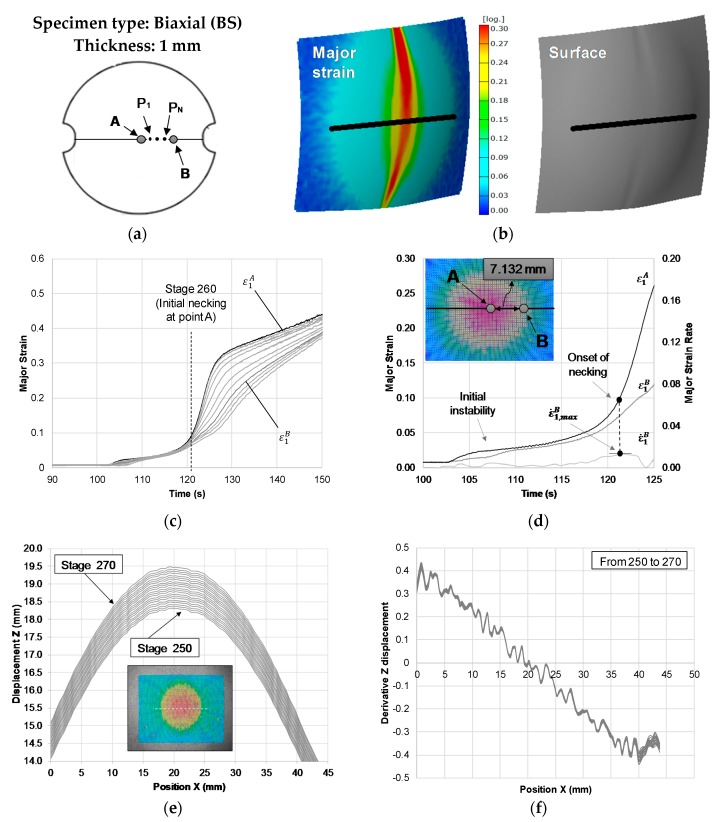
Application of the methodology to obtain the onset of necking strains for a biaxial strain (BS) Nakajima specimen of PC with 1 mm of thickness. (**a**) The geometry of the specimens and identification of the section perpendicular to the failure region and selected points along its length. (**b**) Major strain (left) and surface shape (right) of the specimens’ failure area obtained by the DIC system. (**c**) Experimental time evolution of the major strain along the points of the selected section. (**d**) Time-dependent approach application. (**e**) Z-displacement along the selected section. (**f**) The spatial derivative of Z-displacement along the selected section.

Figure 4 presents the application of the two approaches to the BS Nakajima specimen of PC with 1 mm of thickness. The time-dependent approach (Figure 4c,d), similar to the previous case (Figure 3), allowed to clearly determine the limit strains at the onset of necking that corresponds to point A strains $\left(\varepsilon_{1,lim},\varepsilon_{2,lim}\right)$ at the time instant $t_{necking}$. 

The flat-valley approach application in the BS Nakajima specimen revealed that the identification of the stage at the onset of necking, through the Z-displacement along a section perpendicular to the failure region (Figure 4e) or through the spatial derivative of the Z-displacement along the selected section (Figure 4f), is not as direct as in previous cases (TS and PS). In these specimens the necking region spreads out over a larger region (compare Figure 3d and Figure 4d), making the local reduction in thickness at the necking zone, compared to the adjacent zones, much smaller. This difficult the identification of the onset of necking through the direct observation of changes at the outer surface topology, for which it would be necessary to increase the resolution of the DIC system (see Figure 4f).

Despite this limitation, as depicted in Figure 4b, it is possible to identify the necking region through the thickness reduction and the surface image obtained by the DIC system, which is also possible to visibly identify the necking region through the thickness reduction and the surface image obtained by the DIC system gives the range of time where the onset of necking can occur.

**Figure 5:**
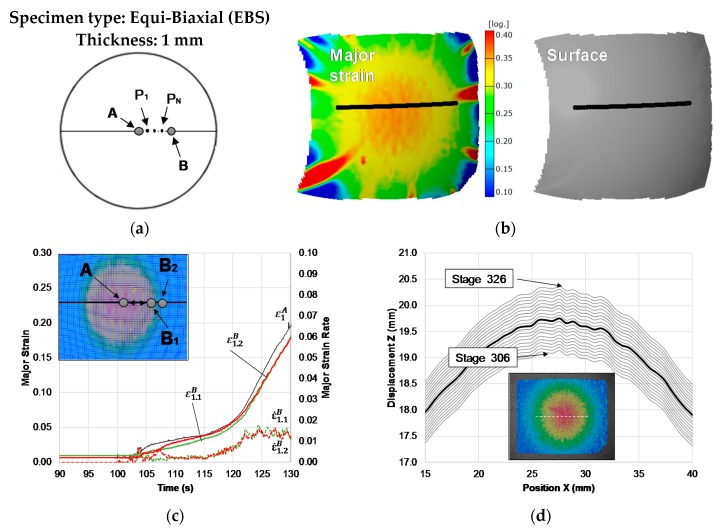
Application of the methodology to obtain the onset of necking strains for an Equi-biaxial strain (EBS) Nakajima specimen of PC with 1 mm of thickness. (**a**) The geometry of the specimens and identification of the section perpendicular to the failure region and selected points along its length. (**b**) Major strain (left) and surface shape (right) of the specimens’ failure area obtained by the DIC system. (**c**) Experimental time evolution of the major strain along the points of the selected section. (**d**) Z-displacement along the selected section.

Figure 5 presents the attempt to apply the two approaches to determine the onset of necking for the EBS Nakajima specimen of PC with 1 mm of thickness. The time-dependent approach revealed that the strain of a series of points along a section perpendicular to the crack showed no ceasing. Additionally, the strain rates for a few points along the section revealed that no local maximum is clearly reached (Figure 5c). Both facts pointed out that a strain localization did not appear in the material under equibiaxial strain.

Similarly, the flat-valley approach application in the EBS Nakajima specimen confirmed that the necking is not a failure mode for PC sheets with 1 mm of thickness under equibiaxial strain (Figure 5b,d).

Table 2 presents the strain pairs at the onset of necking determined by the two approaches, the time-dependent and the flat-valley, for the Nakajima specimens considered in the experimental work plan (Table 1). The obtained necking strain pairs for the TS and PS specimens obtained for the two approaches are almost coincident, for all the three specimens considered for each of the geometries. The maximum deviation in the average necking strain pairs is 12%. 

The results presented in Table 2 for the BS Nakajima specimens revealed that the time-dependent approach allowed to clearly identify the stage at which the onset of necking occurs, while the flat-valley approach allowed to identify a stage interval at which the onset of necking can occur. All the BS specimens tested verified that the stage interval of the flat-valley approach contains the determination of the onset of necking stage identified by the time-dependent approach.

Results show that the application of the time-dependent approach is appropriate to define the time instant at the onset of necking and the corresponding strain pair, for the TS, PS and BS geometries. The flat-valley approach clearly identifies the onset of necking instant for the TS and PS Nakajima specimens, whilst for the BS Nakajima specimens, the result is given by a time interval where the onset of necking can occur. 

The necking strain pairs obtained with this methodology correspond to very low strains, this corroborates that the CGA previously used by Silva et al. [8] and Marques et al. [11], is not appropriate to determine the necking strain pairs, because it is not sensitive enough to identify the onset of necking. 

### 4.2. Strain Paths Evolution Analysis

**Figure 6:**
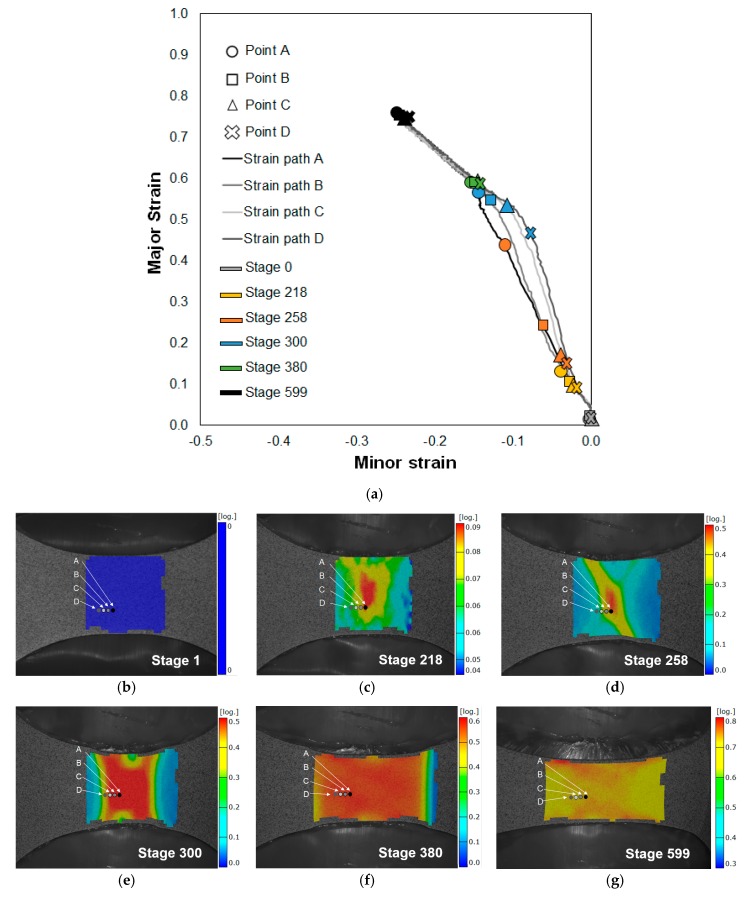
(**a**) Strain path evolution along a section perpendicular to the crack of a TS specimen for different stages through the Nakajima test. Measured major strains obtained by the DIC system for several stages: (**b**) No deformation; (**c**) onset of necking; (**d**,**e**) steadily neck propagation; (**f**) ceasing of neck propagation and (**g**) fracture.

Figure 6a presents the evolution of the strain paths for a series of points along a section perpendicular to the crack of a TS specimen—the strain values were obtained from the DIC system.

On the initial stage (Figure 6b), all the strains are zero, which corresponds to the absence of deformation. With the application of the load, the deformation starts until the onset of necking on point A is reached, stage 218 (Figure 6c), with the prosecution of the loading application the necking propagates steadily along the length of the specimen (Figure 6d,e). When the propagation of the necking ceases, all the strains along the section of the specimen have the same value, stage 380 (Figure 6f). Thereafter the strain field remains nearly homogeneous, increasing its value until the appearance of a crack yielding the final fracture, stage 599 (Figure 6g).

**Figure 7:**
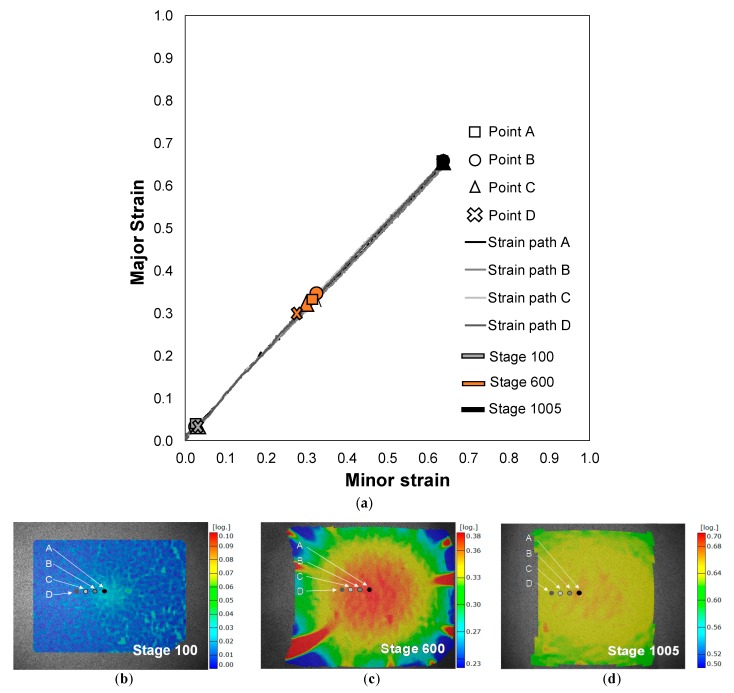
(**a**) Strain path evolution along a section perpendicular to the crack of an EBS specimen for different stages through the Nakajima test. Measured major strains obtained by the DIC system for several stages: (**b**) Initial deformation, stage 100; (**c**) stage 600 and (**d**) stage 1005.

A similar analysis of the strain path was performed for a series of points along a section perpendicular to the crack of an EBS specimen. Figure 7a presents the evolution of the strain paths obtained from the DIC system. 

On an initial stage (Figure 7b) all the strains are near zero, with the application of loading the deformation starts without the occurrence of localization of the deformation on the centre zone of the specimen (Figure 7c), which undergoes under an equibiaxial strain state. The localized deformation (coloured in red in Figure 7c) outside the central area of the specimen is related with the fact that the material outside the central zone is not supported by the punch and develops necks under biaxial conditions, but not under equibiaxial conditions. 

The strain paths of the selected points along a section perpendicular to the crack (Figure 7a) are linear and show no localization in the deformation, this behaviour confirms the results presented in Figure 5, that there is no necking occurrence under equibiaxial conditions for the EBS specimens.

### 4.3. Formability Limit by Fracture

The methodology to determine the fracture strain pairs in metal sheet is based on the measurement of the fracture thickness of the specimens, as described in Section 3.2. This procedure, adapted by considering the local strain ratio β* at the last stages, was applied to the PC sheets with 1 mm of thickness for the four different Nakajima specimen geometries. The fractographies of the specimens were obtained with a NIKON^®^ Stereo Microscope SMZ800N, with a magnification of ×15 and ×30, and allowed the measurement of the fracture thickness.

**Figure 8:**
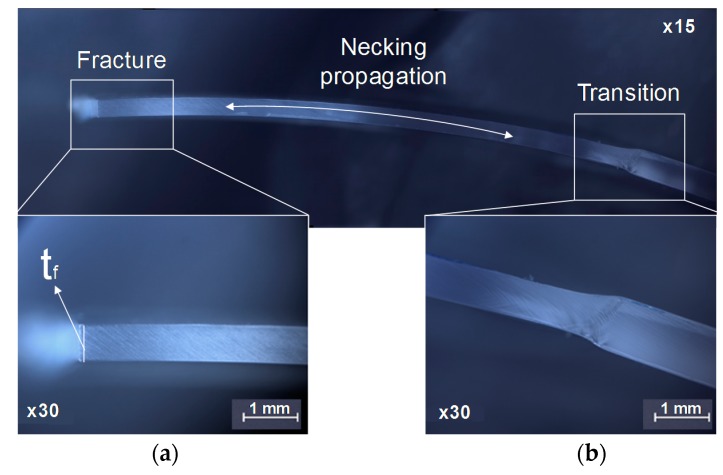
Fractography of a PT Nakajima specimen with 1 mm of thickness. Magnification of: (**a**) The fracture zone and (**b**) the transition zone.

Figure 8 presents the fractographies for a TS Nakajima specimen with 1 mm of thickness, where three different areas are clearly identified: Transition zone, necking propagation zone and fracture zone. The transition zone (Figure 8b) corresponds to the transition between the necked area and the elongated zone, and the fracture zone (Figure 8a) corresponds to where the fracture of the specimen occurs. In between these two zones, it is visible the propagation of the necking zone, typical of polymers when loaded in tension. 

The fracture strain pairs for 1 mm thickness PC sheet were obtained by measuring the fracture thickness from the fractographies of the fractured specimens (Figure 8a) in accordance with the methodology presented in Section 3.2.

The modulus of elasticity of the PC sheets is typically very low, around 2.1 GPa, which produces a significant elastic recovery of the material after fracture. Due to this, the last strain pairs obtained by the DIC system (referred as FFL-DIC) are going to be considered and compared with the fracture strains obtained by the adapted methodology presented in Section 3.2 usually applied to sheet metals (referred as FFL-thickness).

### 4.4. Formability Limits in Principal Strain Space

The formability limits by necking (FLC) and by fracture (FFL) were determined by means of Nakajima tests using the methodologies previously described in Section 3.

**Figure 9:**
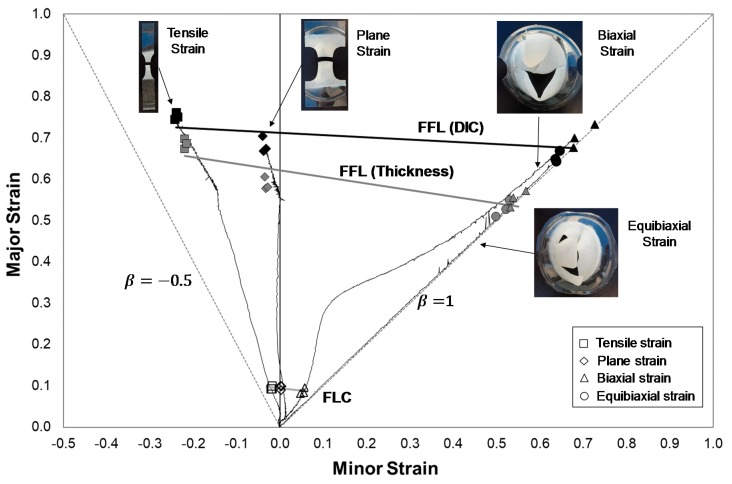
Formability limits by necking (FLC) and by Fracture (FFL) for PC sheets with 1 mm of thickness obtained by means of Nakajima specimens in the tensile, plane, biaxial and equibiaxial strain. The open markers correspond to failure strains by necking, the grey filled marker and black filled markers corresponds to failure strains by fracture obtained measuring the final thickness of the specimens and considering the last strain pairs of the DIC system, respectively.

Figure 9 presents the formability limits and the corresponding failure strains for polycarbonate (PC) sheets with 1 mm of thickness in principal strain space. The determination of the forming limit curve followed the methodology presented in Section 3.1 based on the methodology used to determine the FLC for sheet metal. The resulted FLC shows a different behaviour from the ones previously determined by Silva et al. [8] and Marques et al. [11], this difference can be related with the use of a DIC system that gives a more accurate definition of the onset of necking, due to the steady propagation of the neck, than the use of circle grid analysis which allow measuring the strains only after fracture which corresponds to the propagated necking deformation for fracture.

The use of the DIC system and the proposed methodology revealed to be adequate to characterize the onset of necking in the PC sheet, where the resulted necking limit (FLC) can be represented by a straight line in opposition to the typical FLC of metal sheets that typically have a V shape.

The determination of the fracture limit line (FFL-thickness) followed the methodology presented in Section 3.2 based on the procedure used to determine the FFL for sheet metal. This methodology was previously used by the authors [8,11] and compared with the fracture strains obtained by means of single point incremental forming (SPIF) parts. The fracture strains obtained by the SPIF tests and the Nakajima tests revealed differences that can be related to the significant elastic recovery or springback that polymers experience. As can be seen, the limit strains according to the FFL-Thickness are well below of those from FFL-DIC, due to the severe elastic recovery after fracture. This result is in accordance with the recent investigation of Edwards et al. [20] of the springback relevance when deforming polymeric sheet by incremental forming. 

## 5. Conclusions

The methodologies used to determine the failure limits in sheet metal were adapted to the polymeric sheet, allowing the successful determination of necking and fracture limits for PC sheet with 1 mm of thickness at room temperature (25 °C). The knowledge of accurate formability limits by necking and fracture is relevant for defining sheet forming processes like, for example, incremental sheet forming where the knowledge of the fracture limits is essential [8,9,10,11].

The application of the methodology involved, for the first time ever, the determination of the necking strains of polymer sheets by means of the time-dependent and flat-valley approaches usually used for sheet metals. The utilization of the DIC was demonstrated to be appropriate to obtain the experimental measurements of the strains, regardless of the necking propagation typical of polymers, compared to other inappropriate techniques like, for example, circle grid analysis [11].

The time-dependent approach revealed adequate to characterize the onset of necking for all the specimens where necking occurred, the TS, PS and BS, with good repeatability of the results for all the specimens for each geometry. While the flat-valley approach had some difficulties in identifying a unique stage for the onset of necking for the BS specimens, with an uncertainty interval of 5/3 s, this interval includes the stage obtained from the time-dependent approach. This difficulty of the flat-valley approach may be related to the necking propagation typical of polymers that in this specimen geometry spreads out over a larger region, and consecutively, a smaller local reduction in thickness at the necking zone which was not possible to obtain with the DIC system resolution available. 

The strain path evolution. Along with time analysis for these geometries presented a localization in accordance with the onset of necking and allowed to understand the necking propagation physics typical of polymers until fracture. 

The EBS specimens revealed that necking did not occur which was confirmed by the application of the two necking methodologies. This result was also confirmed by the strain path evolution, along with time analysis where a linear strain path with no localization in the deformation occurred for the EBS specimens.

The methodology used to determine the fracture strains in sheet metal was directly applied to polymeric sheet, and it was verified that the fracture strain pairs for 1 mm thickness PC sheet were obtained by measuring the fracture thickness from the fractographies of the fractured specimens were lower than the last strain pairs obtained by the DIC system on the stage previous to fracture. This phenomenon is due to the low modulus of elasticity of the PC sheets, which produces a significant elastic recovery of the material after the fracture. Thus, the methodology to determine the fracture strains in the polymeric sheet should consider the last strain pairs obtained by the DIC system.

The resulted necking limit (FLC) for the PC sheet can be represented by a straight line in opposition to the typical FLC V shape for sheet metals [33].

The determination of the fracture forming limit was initially performed directly from the sheet metals methodology [12]. It was verified that the resulting major strains from the thickness measurements were lower than the strains from the DIC on the instant before fracture; thus, the later were the ones considered to define the FFL. The resulted fracture limit, FFL (DIC), for the PC sheet can be approximated with a straight line, as was previously stated by previous works by the authors [10,11].

## Figures and Tables

**Table 1:** Summary of the experimental work plan for PC specimens.

Test Geometry	Specimen		Operating Conditions	
	Geometry	Dimensions (mm)	Blank Holder Force (kN)	Velocity (mm/min)
TS	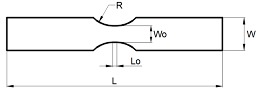	L=200 W=30 $L_{0}=5$ $W_{0}=15$ R=25	30	60
PS	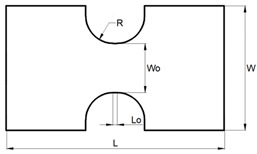	L=200 W=114 $L_{0}=4$ $W_{0}=45$ R=25	40	
BS	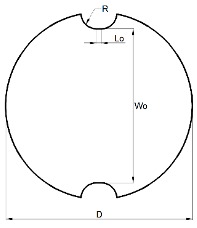	$L_{0}=5$ $W_{0}=150$ R=25 *D* = 182	60	
EBS	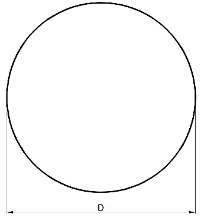	*D* = 182	60	

**Table 2:** Summary of the experimental work plan for PC specimens.

Test Geometry	Specimen	Necking Determination Approaches					
		Time-Dependent			Flat-Valley		
		Stage	$\varepsilon_{1lim}$	$\varepsilon_{2lim}$	Stage	$\varepsilon_{1lim}$	$\varepsilon_{2lim}$
TS	I	251	0.100	−0.019	252	0.106	−0.020
	II	218	0.093	−0.019	220	0.104	−0.024
	III	208	0.093	−0.022	210	0.105	−0.026
PS	I	247	0.099	0.003	249	0.109	0.002
	II	274	0.090	0.003	271	0.081	0.002
	III	243	0.010	0.001	244	0.105	0.000
BS	I	260	0.095	0.057	250–270	–	–
	II	217	0.084	0.055	207–227	–	–
	III	218	0.081	0.047	208–228	–	–
